# Evaluating Hemoglobin Thresholds for Blood Transfusions in Oncology Patients Admitted to the Intensive Care Unit

**DOI:** 10.14740/jh2178

**Published:** 2026-04-06

**Authors:** Barath Prashanth Sivasubramanian, Abijha Boban, Andrew Strike, Moyan Sun, Ania Izabela Rynarzewska, Hardeep Singh, Dhaval Patel

**Affiliations:** a Internal Medicine, Northeast Georgia Medical Center, Gainesville, GA, USA; b Georgia College and State University, Milledgeville, GA, USA; c GME Research, Northeast Georgia Medical Center, Gainesville, GA, USA; d Critical Care Medicine, Northeast Georgia Medical Center, Gainesville, GA, USA

**Keywords:** Transfusion, ICU, Mechanical ventilation, Mortality, Survival

## Abstract

**Background:**

In the intensive care unit (ICU), up to 90% of patients develop anemia during their stay. However, evidence regarding transfusion practices in oncology patients requiring ICU-level care is limited. This study aimed to compare mortality, survival rate, and readmissions across three hemoglobin thresholds of transfusion (low < 7 g/dL, intermediate 7–8 g/dL, and high > 8 g/dL) among these patients.

**Methods:**

A retrospective analysis of 561 patients with cancer admitted to the ICU who received blood transfusions from 2017 to 2023 was performed. Univariate and multivariate analyses were utilized to compare three hemoglobin thresholds of transfusion. A P ≤ 0.05 was considered significant.

**Results:**

Of 561 patients, the transfusion burden was greater in the low threshold cohort (46.6%), followed by intermediate (29.3%) and high (24.1%) thresholds. The low threshold cohort required a longer duration of mechanical ventilation compared to the high threshold (P ≤ 0.03). The readmission rate was highest in the low threshold cohort compared to the others (30-day: 23.4% vs 11% vs 16.3%; 90-day: 3.1% vs 1.2% vs 2.2%). Mortality risk was elevated in patients transfused at high thresholds compared with those transfused at low thresholds (odds ratio (OR), 1.893; 95% confidence interval (CI), 1.093–3.281; P < 0.05), and mortality did not differ between the low and intermediate thresholds (P > 0.05). The intermediate threshold showed the highest survival probability, and the high threshold had the worst survival.

**Conclusions:**

In patients with malignancy admitted to the ICU, transfusions administered at levels < 7 g/dL were associated with a greater transfusion burden, longer mechanical ventilation, and higher 30- and 90-day readmissions. The high threshold was associated with poor survival. These findings highlight the need for prospective studies in ICU oncology on the blood transfusion threshold.

## Introduction

In the intensive care unit (ICU), up to 90% of patients develop anemia during their stay. In the absence of active bleeding, transfusion thresholds for patients with stable comorbidities typically remain at or below 7 g/dL [1–4]. A liberal threshold of 8 g/dL is recommended for patients with acute coronary syndrome [1, 5, 6]. In those with organ dysfunction, transfusion decisions should be individualized based on a risk–benefit assessment [1–4]. The high thresholds (< 10 g/dL) increase the risk of cardiovascular death by approximately 2% in those transfused. Risks from transfusions are higher in patients with cancers when they require ICU-level care [1–4].

It is estimated that 5% of patients with cancer experience a critical illness. These patients account for approximately 15% of all ICU admissions [7]. Anemia in these patients can arise from various factors, including malnutrition related to malignancy, bleeding and coagulopathy, bone marrow dysfunction due to tumor infiltration, primary marrow failure, chemotherapy-induced myelosuppression, surgical blood loss, and chronic inflammation [8–11]. Patients often require multiple transfusions, and anemia is a common cause of hospitalization [12].

However, optimal transfusion practices in cancer remain undefined. This is due to the absence of well-designed prospective studies and the limited sample [3, 4, 12]. Large randomized controlled trials compare restrictive and liberal transfusion thresholds, but these studies do not explicitly study ICU patients [13, 14]. Randomized studies have employed varying transfusion thresholds to define restrictive and liberal transfusion strategies. Prior studies showed no significant differences in mortality between these thresholds. The absence of a clearly defined cutoff across the different trials challenges the translation of these findings into real-world clinical practice [15, 16]. We hypothesize that a liberal transfusion strategy can have better patient-related outcomes [17].

This study aimed to compare mortality, survival, and readmission across three transfusion thresholds (hemoglobin < 7 g/dL, 7–8 g/dL, and > 8 g/dL) in cancer patients admitted to the ICU.

## Materials and Methods

### Study design, population, data collection, and sampling

A retrospective analysis was performed on 561 patients aged 18 years and older with a history of cancer, who were admitted to the ICU at Northeast Georgia Medical Center between 2017 and 2023. Eligible patients received at least one unit of blood transfusion, while those with acute blood loss were excluded. Data were collected via chart abstraction from the health system’s EPIC electronic medical record (EMR) database covering January 2017 to December 2023. Of the patients admitted to the ICU, the five most prevalent cancers were identified and included in the study. They were leukemia, lymphoma, lung cancer, gastrointestinal cancer, and brain cancer. Extracted variables included demographic information, transfusion details, comorbidities (diabetes, chronic kidney disease, chronic obstructive pulmonary disease (COPD), and cardiovascular disease), and complications (respiratory failure, heart failure, myocardial infarction, sepsis, mechanical ventilation, altered mental status, and acute kidney injury). Demographic and clinical data were extracted from the EMR at the time of the patient encounter, with clinical variables identified using the International Classification of Diseases, Tenth Revision (ICD-10) codes. Data were deidentified before analysis, and patients with missing values were excluded using listwise deletion.

### Ethical approval and informed consent

The study protocol was reviewed and approved by the Institutional Review Board (IRB) at Brenau University. Given the retrospective nature of the study, the use of deidentified data, and the absence of more than minimal risk to participants, the requirement for informed consent was waived by the IRB. This study was conducted in compliance with the ethical standards of the responsible institution on human subjects as well as with the Helsinki Declaration [18, 19].

### Statistical analysis

Multiple statistical analyses were conducted to address the research questions. To determine the association between the threshold levels and individual categorical outcomes, a Chi-square crosstabulation test was conducted with a Bonferroni adjustment to compare differences between threshold levels. To assess the difference between the threshold and contributive variables, an analysis of variance and a non-parametric analysis were conducted. Finally, to determine the association with mortality between the threshold levels and key predictor variables, a logistic regression was conducted. The variables were identified based on their significance in binary tests, and regression models were constructed accordingly. The results of regression analysis suggest that the variables included in the equation perform better in the outcome than the baseline model. Kaplan–Meier survival analysis was conducted as a time-to-event analysis to compare survival probabilities across threshold levels [20, 21]. All statistical tests were conducted using SPSS, version 29.

### Outcomes

The primary outcomes of this study were in-hospital mortality and survival probability among cancer patients admitted to the ICU, compared across three transfusion thresholds defined by hemoglobin levels at transfusion (< 7 g/dL, 7–8 g/dL, and > 8 g/dL). Mortality was assessed during the index hospitalization, and survival was evaluated using time-to-event analysis. The survival probability was reported as percentages from the time they received the first transfusion to mortality [20, 21].

Secondary outcomes included hospital readmission within 30, 60, and 90 days following discharge from the index ICU admission. Exploratory (*post hoc*) outcomes included duration of mechanical ventilation and length of ICU stay, measured as total ventilator days and total ICU days during the index hospitalization, respectively. All outcomes were analyzed across the three transfusion threshold groups.

## Results

### Characteristics of the study population

Of the 1,988 ICU patients with a cancer diagnosis, only 561 received blood transfusions and were stratified into three transfusion thresholds: low (< 7 g/dL; 46.6%), intermediate (7–8 g/dL; 29.3%), and high (> 8 g/dL; 24.1%). The average age of patients transfused at the low threshold was 64.97 years, at the intermediate threshold 67.09 years, and at the high threshold 68.50 years. Males were more frequently transfused at the high threshold (low 52.1% vs intermediate 62.2% vs high 65.9%), while females were more commonly transfused at the low threshold (47.9% vs 37.8% vs 34.1%). American White patients were more frequently transfused at a high threshold (86.2% vs 86.6% vs 94.1%), whereas Black patients (8.0% vs 6.1% vs 3.7%) and Hispanic patients were more commonly transfused at the low threshold (4.6% vs 4.3% vs 2.2%). Leukemia (20.7% vs 19.5% vs 24.3%) and lung cancer (34.1% vs 34.1% vs 38.2%) predominated at higher thresholds, lymphoma (16.1% vs 21.3% vs 14.0%) at the intermediate threshold, and gastrointestinal cancer (29.9% vs 26.2% vs 25.7%) at the low threshold. Table 1 shows the sociodemographic characteristics and the type of malignancy in the study population.

**Table 1:** Sociodemographic Characteristics and Type of Malignancy in the Study Population

	Transfusion threshold			
	Hb < 7 (n = 261)	Hb 7–8 (n = 164)	Hb > 8 (n = 135)	Total (n = 560)
Age				
Average	64.97	67.09	68.50	66.44
Gender				
Male				
Count	136	102	89	327
% within transfusion threshold	52.1%	62.2%	65.9%	58.4%
% of total	24.3%	18.2%	15.9%	58.4%
Female				
Count	125	62	46	233
% within transfusion threshold	47.9%	37.8%	34.1%	41.6%
% of total	22.3%	11.1%	8.2%	41.6%
Race				
Other				
Count	15	12	3	30
% within transfusion threshold	5.7%	7.3%	2.2%	5.4%
% of total	2.7%	2.1%	0.5%	5.4%
White				
Count	225	142	127	494
% within transfusion threshold	86.2%	86.6%	94.1%	88.2%
% of total	40.2%	25.4%	22.7%	88.2%
Black				
Count	21	10	5	36
% within transfusion threshold	8.0%	6.1%	3.7%	6.4%
% of total	3.8%	1.8%	0.9%	6.4%
Ethnicity				
Non Hispanic				
Count	249	157	132	538
% within transfusion threshold	95.4%	95.7%	97.8%	96.1%
% of total	44.5%	28.0%	23.6%	96.1%
Hispanic				
Count	12	7	3	22
% within transfusion threshold	4.6%	4.3%	2.2%	3.9%
% of total	2.1%	1.3%	0.5%	3.9%
Type of malignancy				
Leukemia				
Count	54	32	33	119
% within transfusion threshold	20.7%	19.5%	24.3%	21.2%
% of total	9.6%	5.7%	5.9%	21.2%
Lymphoma				
Count	42	35	19	96
% within transfusion threshold	16.1%	21.3%	14.0%	17.1%
% of total	7.5%	6.2%	3.4%	17.1%
Brain cancer				
Count	3	2	5	10
% within transfusion threshold	1.1%	1.2%	3.7%	1.8%
% of total	0.5%	0.4%	0.9%	1.8%
Lung cancer				
Count	89	56	52	197
% within transfusion threshold	34.1%	34.1%	38.2%	35.1%
% of total	15.9%	10.0%	9.3%	35.1%
Gastrointestinal cancer				
Count	78	43	35	156
% within transfusion threshold	29.9%	26.2%	25.7%	27.8%
% of total	13.9%	7.7%	6.2%	27.8%

Hb: hemoglobin.

Patients with cardiovascular disease were most transfused at the high thresholds (19.1%), those with COPD at the intermediate threshold (36.6%), and those with chronic kidney disease were equally transfused at both the low and intermediate thresholds (34.1% each). Patients with diabetes were most transfused at the low thresholds (36.4%). Patients with sepsis (51.0%), acute kidney injury (49.8%), respiratory failure (59.8%), heart failure (36.8%), and altered mental status (11.9%) were most transfused at the low thresholds. Cardiac arrest (11.0%), mechanical ventilation (52.2%), and myocardial infarction (16.9%) were transfused more frequently at the high threshold. Table 2 shows the comorbidities and complications of the study population. The proportion of patients requiring multiple transfusions was highest at the low threshold (76.2%) and lowest at the intermediate threshold (56.7%). Among patients who experienced mortality, the transfusion burden was 19.9% at the low thresholds, 12.8% at the intermediate thresholds, and 25.7% at the high thresholds. Table 3 shows the transfusion burden and the mortality of the study population.

**Table 2:** Comorbidities and Complications of the Study Population

	Transfusion threshold			
	Hb < 7 (n = 261)	Hb 7–8 (n = 164)	Hb > 8 (n = 135)	Total (n = 560)
Comorbidities				
Cardiovascular disease				
Count	32	24	26	82
% within transfusion threshold	12.3%	14.6%	19.1%	14.6%
% of total	5.7%	4.3%	4.6%	14.6%
Peripheral vascular disease				
Count	17	14	9	40
% within transfusion threshold	6.5%	8.5%	6.6%	7.1%
% of total	3.0%	2.5%	1.6%	7.1%
COPD				
Count	88	60	48	196
% within transfusion threshold	33.7%	36.6%	35.3%	34.9%
% of total	15.7%	10.7%	8.6%	34.9%
Chronic kidney disease				
Count	89	56	41	186
% within transfusion threshold	34.1%	34.1%	30.1%	33.2%
% of total	15.9%	10.0%	7.3%	33.2%
Diabetes				
Count	95	45	41	181
% within transfusion threshold	36.4%	27.4%	30.1%	32.3%
% of total	16.9%	8.0%	7.3%	32.3%
Complications				
Sepsis				
Count	133	59	51	243
% within transfusion threshold	51.0%	36.0%	37.5%	43.3%
% of total	23.7%	10.5%	9.1%	43.3%
Acute kidney injury				
Count	130	73	65	268
% within transfusion threshold	49.8%	44.5%	47.8%	47.8%
% of total	23.2%	13.0%	11.6%	47.8%
Respiratory failure				
Count	156	74	68	298
% within transfusion threshold	59.8%	45.1%	50.0%	53.1%
% of total	27.8%	13.2%	12.1%	53.1%
Cardiac arrest				
Count	18	9	15	42
% within transfusion threshold	6.9%	5.5%	11.0%	7.5%
% of total	3.2%	1.6%	2.7%	7.5%
Heart failure				
Count	96	54	49	199
% within transfusion threshold	36.8%	32.9%	36.0%	35.5%
% of total	17.1%	9.6%	8.7%	35.5%
Altered mental state				
Count	31	8	11	50
% within transfusion threshold	11.9%	4.9%	8.1%	8.9%
% of total	5.5%	1.4%	2.0%	8.9%
Mechanical ventilator requirement				
Count	89	72	71	232
% within transfusion threshold	34.1%	43.9%	52.2%	41.4%
% of total	15.9%	12.8%	12.7%	41.4%
Acute respiratory distress syndrome				
Count	18	6	8	32
% within transfusion threshold	6.9%	3.7%	5.9%	5.7%
% of total	3.2%	1.1%	1.4%	5.7%
Pulmonary edema				
Count	20	12	7	39
% within transfusion threshold	7.7%	7.3%	5.1%	7.0%
% of total	3.6%	2.1%	1.2%	7.0%
Myocardial infarction				
Count	33	25	23	81
% within transfusion threshold	12.6%	15.2%	16.9%	14.4%
% of total	5.9%	4.5%	4.1%	14.4%

Hb: hemoglobin; COPD: chronic obstructive pulmonary disease.

**Table 3:** Transfusion Burden and Mortality Rate of the ICU Patients

	Hb < 7 (n = 261)	Hb 7–8 (n = 164)	Hb > 8 (n = 135)	Total (n = 560)
Multiple transfusions				
Count	199	93	93	385
% within transfusion threshold	76.2%	56.7%	68.4%	68.6%
% of total	35.5%	16.6%	16.6%	68.6%
Transfusion complications				
Count	1	0	0	1
% within transfusion threshold	0.4%	0.0%	0.0%	0.2%
% of total	0.2%	0.0%	0.0%	0.2%
Mortality				
Count	52	21	35	108
% within transfusion threshold	19.9%	12.8%	25.7%	19.3%
% of total	9.3%	3.7%	6.2%	19.3%

ICU: intensive care; Hb: hemoglobin.

### Mortality and survival across transfusion threshold levels

**Figure 1:**
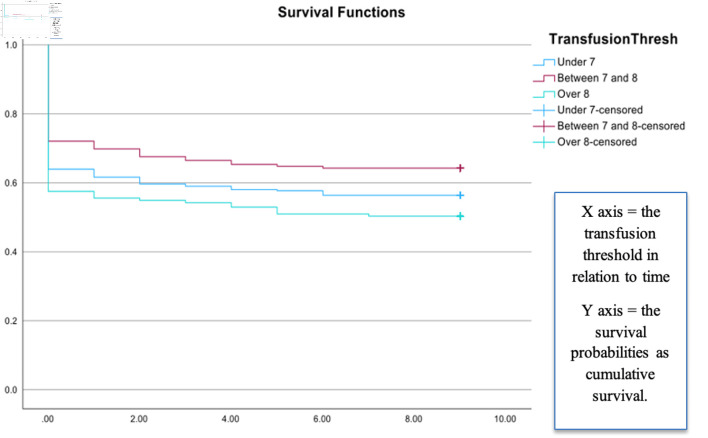
Survival analysis of the ICU patients. Kaplan–Meier survival analysis was conducted to compare survival probabilities across threshold levels, and a statistically significant effect of threshold level on survival was observed (Chi-square = 6.746, df = 2, P < 0.05). The X axis indicates transfusion threshold in relation to time (1 unit of measure = 3 months interval with 0 being 0 to 3 months, 1 being 3 to 6 months, etc.); and the Y axis indicates the survival probabilities (0 to 1 as a proportion value). The lower threshold had a survival probability of 56.4%, and the intermediate threshold had the highest survival of 64.2%. Meanwhile, the higher threshold of 8 or more had the lowest survival of 50.3%.

The results of regression analysis suggest that the variables included in the equation (hemoglobin threshold, age, sepsis, respiratory failure, and days on the ventilator) better predicted the outcome than the baseline model (Chi-square = 96.44, df = 6, P < 0.001). The low threshold level was found to be significant (P = 0.01). The odds of mortality were 89.3% higher among patients transfused at the high thresholds than at the low threshold (odds ratio (OR) = 1.893; 95% confidence interval (CI), 1.093–3.281; P < 0.05). Table 4 shows the results of multivariate analysis. Kaplan–Meier survival analysis was conducted to compare survival probabilities between each threshold level, and a statistically significant impact of threshold levels on survival was noted (Chi-square = 6.746, df = 2, P < 0.05). The low threshold had a survival probability of 56.4% and the intermediate threshold showed the highest survival of 64.2%. Meanwhile, the higher threshold had the lowest survival of 50.3%. Figure 1 shows the results of the survival analysis.

**Table 4:** Multivariate Analysis of Mortality

	Adjusted odds	95% CI		Wald	df	Significance
		Lower	Upper			
Hemoglobin < 7	Reference			8.138	2	0.017
Hemoglobin 7–8	0.783	0.430	1.427	0.637	1	0.425
Hemoglobin > 8	1.893	1.093	3.281	5.180	1	0.023
Age	1.019	0.998	1.040	3.204	1	0.073
Sepsis	2.185	1.341	3.563	9.834	1	0.002
Respiratory failure	5.063	2.746	9.335	27.006	1	< 0.001
Ventilator days	1.048	1.015	1.082	8.359	1	0.004
Constant	0.012			33.090	1	< 0.001

CI: confidence interval.

### Readmission at 30 days, 60 days, and 90 days

Based on a crosstabulation test, it can be concluded that there is an association between readmission rates and the threshold levels (P ≤ 0.041), as shown in Table 5. Readmission rates were highest among patients transfused using intermediate thresholds (82.9%, z = 1.2) and lowest among those transfused using the low threshold (69%, z = –1.1). Patients transfused at the low threshold had the highest readmissions at 30 days (23.4%, z = 2) and 90 days (3.1%, z = 0.8); those transfused at intermediate thresholds had the lowest 30-day (11%, z = –2.1) and 90-day (1.2%, z = –0.9) readmission rates. In no readmission and readmission within the first 30 days, the low and the intermediate thresholds were statistically different from each other, but the high transfusion threshold was not statistically different from the other two. Results of readmission are shown in Table 5.

**Table 5:** Readmission Rates of the ICU Patients

	Transfusion threshold			
	Hb < 7 (n = 261)	Hb 7–8 (n = 164)	Hb > 8 (n = 135)	Total (n = 560)
No readmission				
Count	180	136	103	419
% within transfusion threshold	69	82.9	76.3	74.8
Standardized residual	–1.1	1.2	0.2	
30-day readmission				
Count	61	18	22	101
% within transfusion threshold	23.4	11	16.3	18
Standardized residual	2	–2.1	–0.5	
60-day readmission				
Count	12	8	7	27
% within transfusion threshold	4.6	4.9	5.2	4.8
Standardized residual	–0.2	0	0.2	
90-day readmission				
Count	8	2	3	13
% within transfusion threshold	3.1	1.2	2.2	2.3
Standardized residual	0.8	–0.9	–0.1	

Based on a crosstabulation test, it can be concluded that there is an association between readmission rates and the threshold levels (Chi-square = 13.105, df = 6, P ≤ 0.041). ICU: intensive care; Hb: hemoglobin.

### Mechanical ventilation duration and length of ICU stay

The mean mechanical ventilation duration was longest in the low transfusion threshold group (mean 2.90 ± standard deviation (SD) 8.37 days) compared with the intermediate (2.14 ± 5.57 days) and high (2.29 ± 4.13 days) threshold groups. However, a non-parametric test (Kruskal–Wallis) revealed a statistical impact of the threshold levels of the days on ventilator (H = 6.65, df = 2, P ≤ 0.036). More specifically, a difference was found between the low and high thresholds adjusted for multiple comparisons (P ≤ 0.03). The mean ICU stay was longer in the low threshold group (6.72 ± 9.90 days vs 5.85 ± 8.01 days vs 5.13 ± 5.67 days). No statistical difference was found between the threshold levels and the ICU days.

## Discussion

Among the 561 patients with a diagnosis of malignancy who were admitted to the ICU and received blood transfusions, the transfusion burden was greatest at the lower hemoglobin threshold (< 7 g/dL) and lowest at the higher threshold (> 8 g/dL). Across all thresholds, the average age of transfused patients was 66.4 years. Notable demographic differences were observed, with male patients tending to receive transfusions at the high threshold. Racial disparities were evident, with White patients (n = 494) more frequently transfused at the high thresholds, while Black and Hispanic patients were more often at the low threshold. However, our study had limited representation from Black (n = 36) and Hispanic (n = 22) patients. The proportion of patients requiring multiple transfusions was the largest among those in the low-threshold group. Patients transfused at the low threshold required mechanical ventilation for the longest duration, had prolonged ICU stays, and experienced the highest 30-day and 90-day readmission rates. Mortality risk was elevated in patients transfused at the high threshold compared with those transfused at the low threshold. No significant difference in mortality was observed between the low and intermediate (7–8 g/dL) thresholds. The intermediate threshold was associated with the best survival probability among the three groups.

In our study, patients with leukemia received transfusions at the high threshold. Short-term red blood cell (RBC) transfusion is commonly used for transient anemia during induction chemotherapy or hematopoietic stem cell transplantation. Transfusion thresholds for hematologic malignancies often align with those established for general critical care patients [22]. However, in the presence of complications, the high thresholds can be used [23]. For patients with solid malignancies such as lung and gastrointestinal malignancies, transfusion strategies are based on disease and treatment status. In the ICU, patients with an advanced stage of disease perioperatively require frequent transfusions [24–26]. In our study, patients with cardiovascular disease and myocardial infarction were frequently transfused at the high threshold. The high threshold of 8 g/dL is recommended for patients with acute coronary syndrome [1, 5, 6] as the mortality rate was higher in those treated with a restrictive strategy (restrictive 9.9% vs 8.3% liberal) [27]. Transfusion thresholds for patients with stable comorbidities typically remain at or below 7 g/dL [1–4]. For patients with COPD, this becomes challenging as they are at a higher risk for decompensation. In our study population, they were commonly transfused at the intermediate threshold. ICU patients have several risk factors that lead to the development of AKI. These patients require an earlier intervention [28]. In our study, these patients were commonly transfused at the low threshold. The decision to transfuse can be made using clinical decision tools, and education could prove useful in the acute setting [29, 30]. Patients with sepsis (51.0%) and altered mental status (11.9%) were commonly transfused at the low threshold. In septic shock, the CHEST society recommends a restrictive transfusion strategy due to no difference in ICU mortality between restrictive and liberal thresholds [26]. Blood transfusion, infection, and longer ICU stays were previously identified to be associated with delirium [31]. We observed an 11.9% incidence of altered mental status during hospital stay. Prospective studies are needed to further examine this finding.

A meta-analysis by Wisnawa et al [15] reported no significant differences in mortality between the different thresholds (heterogeneity of I^2^ = 0%). However, randomized studies have employed varying transfusion thresholds to define restrictive and liberal transfusion strategies. This makes it challenging to translate these findings into real-world clinical practice [15]. Oncology patients requiring ICU-level care often have an evolving clinical picture. The results of our study indicate that the high threshold was associated with higher mortality and poorer survival. Patients with mechanical ventilation requirements and an episode of cardiac arrest were frequently transfused at the high threshold. Increased dependence on the ventilator is an independent predictor of mortality. Future prospective studies are required to evaluate the benefits of transfusion in those who are mechanically ventilated, in shock, and have end-of-life needs [32, 33].

This study has several limitations. The cancer status of patients, such as active disease or remission status, could not be determined. Key clinical details such as the etiology of anemia, indication for transfusion, transplantation status, receipt of active treatment, timing of the last chemotherapy dose, and goals of care were not available. The need for multiple transfusions was identified. However, the interval between transfusions could not be assessed. Long-term outcomes, including changes in functional status and quality of life, were not captured. The retrospective design prevents any determination of causality, and patients with missing hemoglobin values were excluded by listwise deletion. The retrospective nature of our study was prone to loss to follow-up of patients and could not be avoided. The analysis was restricted to ICU patients and included only the five most common cancers at our institution, resulting in a less homogeneous study population. Larger prospective studies are needed to confirm these findings and determine optimal transfusion strategies.

### Conclusions

The findings of our retrospective study indicate that transfusions administered at hemoglobin levels < 7 g/dL were associated with greater transfusion burden, longer mechanical ventilation duration, and higher 30- and 90-day readmission rates. In contrast, transfusions administered at hemoglobin levels > 8 g/dL were associated with increased mortality, while survival was greater among patients transfused at the low threshold < 7 g/dL, with the intermediate 7–8 g/dL threshold associated with the highest survival probability. These findings highlight the need for prospective studies to optimize transfusion threshold in critically ill oncology patients.

## Data Availability

The authors declare that data supporting the findings of this study are available within the article.
